# Solar Radiation Determines Site Occupancy of Coexisting Tropical and Temperate Deer Species Introduced to New Zealand Forests

**DOI:** 10.1371/journal.pone.0128924

**Published:** 2015-06-10

**Authors:** Robert B. Allen, David M. Forsyth, Roy K. J. Allen, Kathrin Affeld, Darryl I. MacKenzie

**Affiliations:** 1 Landcare Research, Lincoln, New Zealand; 2 Arthur Rylah Institute for Environmental Research, Department of Environment, Land, Water and Planning, Heidelberg, Victoria, Australia; 3 Independent Researcher, Galatea, Murupara, New Zealand; 4 Proteus Wildlife Research Consultants, Dunedin, New Zealand; University of Sydney, AUSTRALIA

## Abstract

Assemblages of introduced taxa provide an opportunity to understand how abiotic and biotic factors shape habitat use by coexisting species. We tested hypotheses about habitat selection by two deer species recently introduced to New Zealand’s temperate rainforests. We hypothesised that, due to different thermoregulatory abilities, rusa deer (*Cervus timorensis*; a tropical species) would prefer warmer locations in winter than red deer (*Cervus elaphus scoticus*; a temperate species). Since adult male rusa deer are aggressive in winter (the rut), we also hypothesised that rusa deer and red deer would not use the same winter locations. Finally, we hypothesised that in summer both species would prefer locations with fertile soils that supported more plant species preferred as food. We used a 250 × 250 m grid of 25 remote cameras to collect images in a 100-ha montane study area over two winters and summers. Plant composition, solar radiation, and soil fertility were also determined for each camera location. Multiseason occupancy models revealed that direct solar radiation was the best predictor of occupancy and detection probabilities for rusa deer in winter. Multistate, multiseason occupancy models provided strong evidence that the detection probability of adult male rusa deer was greater in winter and when other rusa deer were present at a location. Red deer mostly vacated the study area in winter. For the one season that had sufficient camera images of both species (summer 2011) to allow two-species occupancy models to be fitted, the detection probability of rusa deer also increased with solar radiation. Detection probability also varied with plant composition for both deer species. We conclude that habitat use by coexisting tropical and temperate deer species in New Zealand likely depends on the interplay between the thermoregulatory and behavioural traits of the deer and the abiotic and biotic features of the habitat.

## Introduction

Understanding why animal species do or do not use a location has long been a fundamental question in ecology [1–5]. In particular, there is debate about the relative importance of abiotic and biotic factors for species distributions and coexistence [6]. Abiotic factors (e.g. climate) are often considered to limit the distribution and coexistence of species at large spatial scales [7, 8], whereas biotic factors (e.g. food availability and competition) are important at a local scale, often interact, and can operate in concert with abiotic factors [6, 7]. It is therefore challenging to distinguish the role of specific abiotic and biotic factors in relation to habitat use and activity [7].

Contemporary habitat use and activity not only reflects the current role of abiotic and biotic factors, but also the influence of evolutionary history [5]. In coevolved communities, species may have competed in the past and evolved different behavioural and/or morphological features that are now manifested through contemporary habitat selection. As a consequence, it can be difficult to distinguish whether contemporary habitat use and activity are the result of current factors or legacies of a coevolved history [5, 9]. An emerging opportunity for understanding how current abiotic and biotic factors alone shape the use of habitat by (and activity of) coexisting species is through studying recent assemblages of introduced species. In such ecosystems, behavioural and/or morphological features are expressed through contemporary habitat selection only in response to current abiotic and biotic factors.

Thirteen ungulate species introduced to New Zealand between the early 1800s and 1910 have established self-sustaining populations [10]. Most of these ungulate species evolved in the Northern Hemisphere temperate zone, with only two species evolving in the tropical zone. Several studies have investigated hypotheses about species coexistence in New Zealand’s introduced ungulate communities. Reduced abundances of Alpine chamois (*Rupicapra rupicapra*) following increases in abundances of Himalayan tahr (*Hemitragus jemlahicus*) were attributed to behavioural avoidance of the latter by the former [11]. Sika deer (*Cervus nippon*) out-compete red deer (*Cervus elaphus scoticus*) in central North Island forests [12, 13], most likely due to the ability of sika deer to digest lower quality forage [14, 15]. More generally, habitat use and activity by ungulates introduced to New Zealand is believed to be largely determined by the abundance of preferred browse plant species (reviews in [16, 17]), the abundance of which are, in turn, influenced by soil fertility and landform [18, 19], and stage of forest development [20].

In this study we used camera traps and site occupancy modelling to evaluate small-scale habitat use and activity by sympatric rusa deer (*Cervus timorensis*) and red deer introduced into New Zealand’s temperate rainforests. Small-scale habitat selection studies can provide insights into the role of specific abiotic and biotic factors [8, 21]. Our study most closely corresponds to selection of habitat components within a home range (third-order selection *sensu* Johnson [22]) and was based upon a population-level-use sampling design (i.e. design I [23]). Camera traps are revolutionising such habitat selection studies because they allow relatively unobtrusive and continuous monitoring at locations of interest [24, 25] and enable imperfect detectability to be accounted for in analyses of site occupancy [26]. Occupancy is a particularly useful metric for evaluating the relative influence of abiotic and biotic factors on the presence of species at a location because multiple covariates, including season and the influence of other species, can be simultaneously accounted for, along with detection probability [27, 28].

We compare occupancy and detection probabilities of rusa deer and red deer in relation to covariates suggested by their biogeographic origins and colonisation patterns. Rusa deer are believed to be native to Indonesia, but have also been introduced to many tropical islands in the Indo-Pacific region, as well as Australia and New Zealand [29]. Rusa deer were introduced to New Zealand in 1907 and are the least widespread deer species, occupying approximately 565 km^2^ in the central North Island [30]. Red deer naturally inhabit most of Europe, parts of western Asia and central Asia, as well as north-western Africa, and they have been introduced widely in the southern hemisphere. Red deer were first introduced into New Zealand in 1851 and are now the most widespread ungulate species, occupying 120,000 km^2^ (ca. 44%) of New Zealand’s mainland [31]. Observations suggest that rusa deer are susceptible to winter conditions in their temperate New Zealand and Australian ranges, with dead rusa deer being observed after periods of cold weather during winter [32, 33]. In contrast, red deer can withstand cold winter conditions, including deep snow [34]. We therefore hypothesised that rusa deer would select relatively warm habitats in winter, particularly those with high direct solar radiation [35]. Adult male rusa deer become viciously aggressive during the winter rut [30, 36], such that red deer may be excluded from locations used by rusa deer during that season. We hypothesised that habitats with a dominance of rusa deer in winter will also have a relatively high proportion of adult male rusa deer and a relatively low proportion of red deer. In summer, climate is not thought to influence habitat use by rusa deer. Our final hypothesis was, therefore, that both rusa deer and red deer would prefer habitats with fertile soils (high soil Nitrogen (N) and Phosphorus (P) availability) and an abundance of preferred food plants in summer. Our combination of camera trap data and site occupancy modelling showed that small-scale habitat use by coexisting rusa deer and red deer likely depends upon the interplay between the traits of the deer species and features of the habitat.

## Material and Methods

### Study area

**Fig 1:**
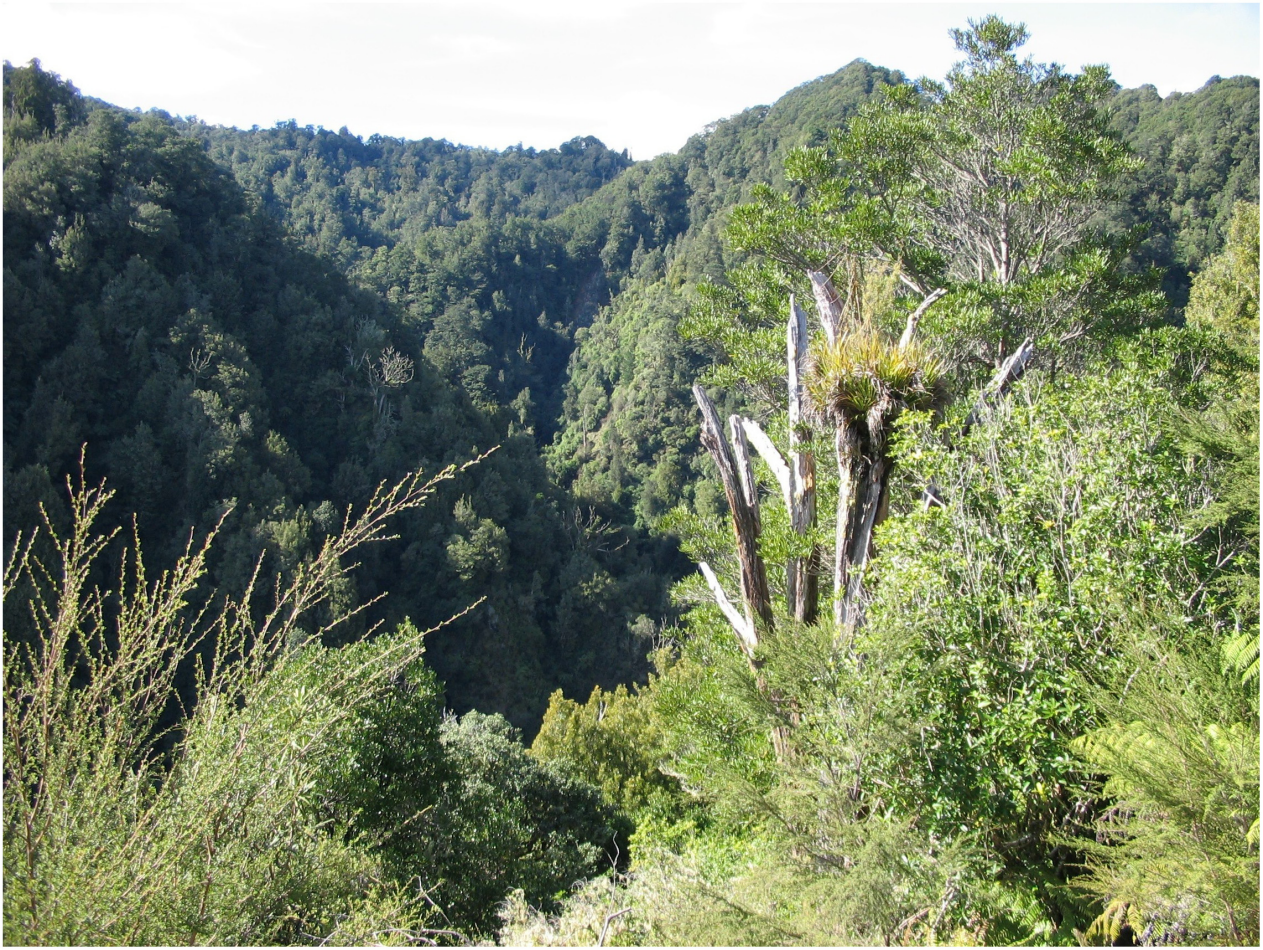
Photograph of Ikawhenua Range, illustrating the topographic diversity and forest cover of our study area. A 400-m elevation gradient occurs from the valley bottoms to ridge crests, forming steep slopes of varying aspects. *Kunzea ericoides*, a tree species dominating previously burnt forest, is shown in the foreground, with trees of *Beilschmiedia tawa* and *Melicytus ramiflorus* dominating the unburnt forest slopes in the background. A grid of camera locations was used to representatively sample the range of sites and determine occupancy by sympatric tropical and temperate deer species.

Data were collected from a 100-ha area of rainforest 300–700 m above sea level in the Ikawhenua Range, Te Urewera, New Zealand (38°20'S 176°50'E). The rainforest is on land administered at the time of this study by the New Zealand Department of Conservation (but now by Te Urewera Board), who gave field study permission under an operating concession to Landcare Research. These forests are on steep slopes with deeply incised valleys formed by small streams (Fig 1). The greywacke parent material (a P-poor, fine-grained sandstone) [37] is overlain by a series of airborne rhyolitic pumice deposits from nearby volcanoes. These produce free-draining, nutrient-poor soils, especially with respect to P [37]. Notwithstanding that, marked variation in soil fertility occurs over short distances (<50 m), and in a nearby topographic gradient a >50-fold variation in soil total P occurs from ridges, through faces and terraces, to gullies [38]. Climate data were available from the Galatea Basin Climate Station (160 m elevation and 10 km south-west of the study area) [39]. The climate is oceanic; winter daily means (±SE) of 8.3 ± 0.8°C and 8.0 ± 0.3°C were measured in 2010 and 2011, respectively, and summer means of 18.9 ± 0.2°C and 16.8 ± 0.2°C were measured in the austral summers of 2010/2011 (hereinafter “2010”) and 2011/2012 (“2011”), respectively. Extreme winter minimums were −3.8°C and −4.8°C in 2010 and 2011, respectively. Winter mean daily precipitation was 4.0 ± 0.9 mm and 2.2 ± 0.4 mm in 2010 and 2011, respectively, and mean daily precipitation was 4.9 ± 1.7 mm and 3.4 ± 1.0 mm in the austral summers of 2010 and 2011, respectively.

Part of the study area is forested with an evergreen angiosperm canopy (>15 m tall) dominated by *Beilschmiedia tawa*, *Weinmannia racemosa* and *Nothofagus fusca*, above which is an emergent canopy (>30 m) of long-lived, slow-growing conifer species, particularly *Dacrydium cupressinum*, *Prumnopitys ferruginea* and *P*. *taxifolia*, as well as the evergreen angiosperm *Metrosideros robusta* along ridge crests [40]. Small trees and shrubs (e.g. *Pseudopanax* spp., *Pseudowintera* spp. and *Coprosma* spp.) and tree ferns (*Cyathea* spp. and *Dicksonia* spp.) occupy the subcanopy (up to 8 m) and can contribute to the canopy in steep gullies. The understorey is dominated by ferns (e.g. *Cyathea* spp., *Asplenium* spp., *Blechnum* spp., *Hymenophyllum* spp. and *Polystichum* spp.) and *Uncinia* spp. Where the forest has been burnt, the angiosperm canopy (ca. 10 m tall) is instead dominated by *Kunzea ericoides* and *Knightia excelsa*, particularly along ridge crests and faces, and *Melicytus ramiflorus* on lower slopes and gullies [35, 41]. The understorey of post-fire vegetation is also dominated by a suite of ferns and *Uncinia* spp., with increased herb and grass dominance where the canopy is open. Areas fenced to exclude ungulates indicate that deer sometimes alter understorey plant species composition of Te Urewera forest sites [42].

### Study species

Rusa deer were released in nearby Galatea, and from 1907 to the 1950s were apparently confined to farmland and adjacent shrublands [43], but by the 1960s they were present in the forested study area [35]. Overall numbers dramatically increased in the early years and were probably highest in the 1950s to 1970s; they then declined in the 1980s and 1990s before increasing again in the 2000s [44]. The decline was largely caused by commercial deer harvesting, and the more recent increase by a reduced level of commercial harvest [44]. Red deer have been present in our study area for over 100 years [40]. The red deer population has probably shown a similar temporal trend to rusa deer over recent decades because red deer were subjected to a similar commercial hunting regime.

**Fig 2:**
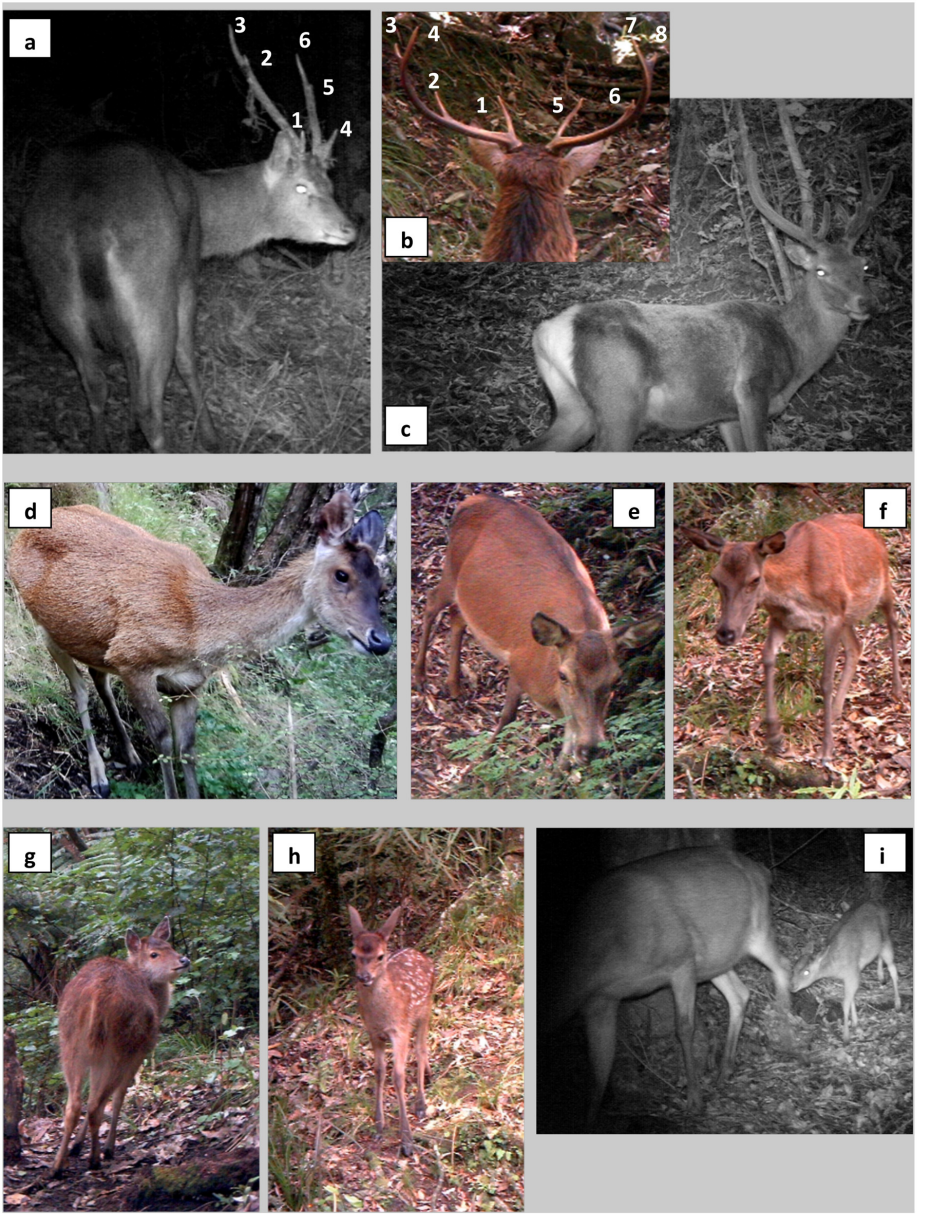
Distinguishing features of rusa deer and red deer. Antlers of mature rusa deer males typically have a maximum of six points and a lyre shape (a), whereas red deer antlers often exceed six points (b). Rusa deer have a long slender tail, slightly bushy towards the tip, and a light-coloured rump patch (a) compared with red deer, which have a short tail and distinct white rump patch (c). The face of rusa deer is shorter, as are the ears, which have rounded tips (d) compared with the red deer’s elongate face and longer ears with pointed tips (e, f). A dark strip of hair along the spine (e) and light-coloured hair on the belly (f) are further characteristics of red deer that are less pronounced in rusa deer (d). White spots are absent on the coat of rusa deer juveniles (g) but present on red deer juveniles (h). Juveniles of both species are defined as being less than two-thirds of the size of the adult (i). Although both rusa deer and red deer have coats with a reddish tinge in summer and a greyish tinge in winter, the reddish tinge is more pronounced in red deer and the greyish tinge more pronounced in rusa deer.

Male rusa deer cast their antlers in December–January, and new antler growth is complete by May [35]. In contrast, male red deer cast their antlers in September and complete antler growth by March [31]. The rusa deer rut starts in mid-July and continues into August. The red deer rut starts in late March and continues through much of April. Rusa deer fawns are typically born in March–April, and red deer fawns are typically born in November–December [31]. There is no evidence for hybridisation between rusa deer and red deer in the wild [45]. The body masses of adult rusa are ca. 90% of those of the taller red deer in the study area (R.K.J. Allen, personal observation), and several morphological features can be used to distinguish the two species in the field (Fig 2) [45]. The coat of red deer has a red tinge, whereas the coat of rusa deer has a grey tinge. A dark strip of hair along the spine, and the light colour of hair on the belly is more pronounced in red deer compared with rusa deer. Rusa deer have a long slender tail, more bushy towards the tip, and no light-coloured rump patch (caudal disc) compared with red deer (which have a short tail and distinct rump patch). The face and ears of rusa deer are shorter than red deer, which have an elongate face and long ears. Rusa deer have ears with rounded tips, whereas red deer ears have pointed tips. The configuration and shape of adult male rusa deer antlers is typically a maximum of six points and a lyre shape, whereas red deer antlers often exceed six points and have a bowl shape. White spots are absent on the coat of juvenile rusa deer, but are present on juvenile red deer (Fig 2).

### Data collection

Digital trail cameras with a passive infra-red movement sensor (Scout Guard DTC-530V, Shenzhen Siyuan Digital Technology Co. Ltd, Shenzhen, Guangdong, China) were used and triggered by deer movement. The camera is activated within one second after detecting movement and automatically takes colour images during daylight hours and black and white images at night using infra-red light-emitting diode no-flash technology (12-m range). Under New Zealand law, animal ethics approval was not required for taking such images. Each camera was programmed to take a three-megapixel image, with a minimum interval between images of 10 s, and to record a date and time stamp on each image. A total of 25 cameras were located (using a Global Positioning System (GPS) device) to form a grid at 250-m intervals, including the boundaries, in the 1 km by 1 km study area. A camera was mounted on a tree trunk (at a height of 1.5 to 2 m) at each grid-point and orientated to cover an image area of 4 m by 8 m containing at least one animal track. Each camera recorded deer over the winter seasons (mid-June to mid-September) and the austral summer seasons (mid-December to mid-March). The Secure Digital (SD) card storage devices in each camera were changed and downloaded at the midpoint and end of each season. This, together with operational tests when the SD cards were changed, reduced the risk of incomplete data capture because of malfunctioning cameras.

Data were obtained by displaying SD card images on a computer screen with zoom capability and recording the following: camera identifier; start and end date of SD card operation; image number; date; time; deer species, age and sex (Fig 2). Age was recorded as “adult” or “juvenile”, based upon size. Juveniles were defined as being less than two-thirds of the size of most adults. Sex of adults was classified as male or female, based upon the presence of antlers or not, with “unknown” recorded for headless images. When there were consecutive images of the same individual within a period of <5 min, these were considered non-independent and only the first image was used in analyses. The camera-trap data for each location were aggregated to a weekly scale, i.e., whether the deer species (and adult male rusa deer) were detected in that 7-d period or not. Common weekly periods were used across all sites, and the number of days during each week when the camera was active was determined.

For each camera location, we recorded vascular plant species composition (plant composition) on each image area by recording species in seven fixed height tiers (0–30 cm; >30 cm–2 m; >2 m–5 m; >5 m–12 m; >12 m–25 m; >25 m; epiphyte) and their respective cover-abundance in each tier using a modified Braun-Blanquet scale (1 = <1%, 2 = 1–5%, 3 = 6–25%, 4 = 26–50%, 5 = 51–75%, 6 = 76–100%) [46]. Cover-abundance scores within each height tier were converted to the midpoint of the percentage cover range for that cover-abundance class, and summed across tiers for each species [47]. This generated an importance value for each species on each vegetation plot, reflecting the volume occupied by each species rather than its projected cover. These species importance values were used as weights in a Bray–Curtis similarity index of plant composition between every pair of camera locations. These similarity indices were then employed in Non-metric MultiDimensional Scaling (NMDS) in PRIMER 5 [48] to position camera locations in two-dimensional space, based upon their distances from one another, where a larger distance represented lower similarity in plant composition. The most important dimension of compositional variation is represented by the NMDS Axis 1 scores for camera locations. NMDS makes few assumptions about the underlying distributions of species in two-dimensional space [48]. All data were standardised to account for differences in location total sample volumes, and fourth-root transformed to give rarer species greater weight in the calculation of similarities [48]. The calculations were performed with 10 random restarts. The two-dimensional ordination (stress value 0.22) and subsequent cluster analysis distinguished five plant communities, although one community was represented by only one camera location. We used a hierarchical agglomerative clustering method that fused camera locations, based on the Bray–Curtis similarity index, into types using group average linking [48]. SIMPER analysis in PRIMER 5 [48] was then used to identify the species that primarily contributed to the discrimination of community types.

Total, direct and diffuse solar radiation transmitted through the forest canopy and arriving at the image area were quantified for each camera location using a hemispherical photograph. These were taken at the centre of each image area with a Nikon COOLPIX digital camera fitted with a 180° hemispherical lens [49]. Photographs were taken on an overcast day, to reduce the effect of glare, at a height of 1.4 m above the ground. To obtain estimates of direct, diffuse and total solar radiation (our measures of warmth at a location), images were analysed using Gap Light Analyzer V2 software [50]. The software accounts for the sun track and estimates solar radiation, from the hemispherical photos, by integrating transmission over all days within each of the summer and winter periods (see [49]). Direct, diffuse and total solar radiation were determined as the total mols m^-2^ day^-1^ transmitted through the canopy. Software was configured for New Zealand solar radiation, and accounted for slope and aspect, but not cloud cover. In addition, a pooled soil sample was collected in winter 2012 from the upper 10 cm of the mineral horizon to characterise soil fertility in the plant rooting zone [38]. Five subsamples were collected systematically within each image area and pooled to give a representative sample of the rooting zone [38], then air dried, sieved (<2 mm) and analysed for Bray 2 available P (available P; μg g^-1^), pH (in water), and percentage total C and N (FP2000 CN analyser; LECO Corp., St Joseph, MI) using methods described by Blakemore et al. [51]. Results are expressed on an oven-dry (105°C) soil basis. High mineral soil C:N ratio indicates low soil N availability [52, 53].

### Occupancy modelling

Inspection of the camera trap data revealed that there were insufficient red deer images to model occupancy of this species, except in summer 2011. We therefore used multiseason occupancy models [28, 54] to evaluate the influence of solar radiation (i.e. total, direct and diffuse solar radiation) and the relative importance of other covariates (i.e. plant composition and soil fertility) on occupancy and detection probabilities of rusa deer among seasons. Year effects were not considered in any models because we had no expectation of between-year differences beyond those that could be explained by our covariates. The parameters estimated using multiseason occupancy models are the probability of rusa deer occupancy at a location and the probability of detection (i.e. at least one image within a week), conditional on rusa deer using a location. Detection probability is thus related to activity. Our multiseason occupancy model assumed that whether rusa deer are present or absent at a location in a given season is independent of whether they were present at that location in the previous season. MacKenzie et al. [28] term this an “implicit dynamics model” because it implicitly assumes that changes in occupancy between seasons are random, although it can be used to investigate the overall occupancy patterns in each season. The influence of covariates on occupancy and detection probabilities was investigated using a procedure analogous to logistic regression or generalised linear models. Importantly, the occupancy modelling framework corrects the logistic regression occupancy coefficients for imperfect detection. The multiseason models were used to assess whether the effects of covariates on occupancy, or detection, varied by season using interaction terms between covariates and season. The square root of direct, diffuse and total solar radiation, and the natural logarithm of soil fertility covariates were used to reduce the potential influence of infrequent, larger values that may have had undue leverage on results when fitting linear relationships. The natural logarithm is commonly used in such circumstances, although we also used the square root transformation (as some solar radiation values were zero and we wished to avoid using a logarithm transformation with an arbitrary offset). A seasonal effect was included in all models for both occupancy and detection. In addition, one covariate at a time was included in each model. When the additional covariate was included in each specific model, the effect was assumed to be either the same in both seasons (i.e. an additive, or “+” model) or different in each season (i.e. an interaction, or “×” model). The same additional covariate was considered for both occupancy and detection. The 59 candidate multiseason occupancy rusa deer models that we confronted with data are listed in S1–S3 Tables.

To determine whether occupancy or detection of adult male rusa deer differed between winter and summer, we used a multistate, multiseason model [55]. This model was used to test our hypothesis that habitats with high rusa deer activity in winter would also have a high proportion of adult male rusa deer. The possible occupancy states in this model were: (1) no rusa deer; (2) rusa deer, but no adult male rusa deer; and (3) adult male rusa deer (and possibly other rusa deer). The camera trap location must be in one of these three occupancy states; for states 2 and 3, rusa deer must be detected at the location. The occupancy-related parameters estimated in this model are the probability of rusa deer being present (i.e. state 2 or 3), and the conditional probability of adult male rusa deer occupancy (the probability that adult male rusa deer are present (i.e. state 3) given that rusa deer are present (i.e. states 2 or 3)). If adult male rusa deer become more widespread in some seasons, relative to the distribution of rusa deer in general, then the conditional probability of adult male rusa deer occupancy would be expected to increase. The multistate, multiseason model also allows the probability of rusa deer detection to differ for different occupancy states; for those locations where adult male rusa deer were present, this model indicates the probability of detecting adult male rusa deer given rusa deer were detected. That is, given rusa deer were detected within a week, what is the probability that (at least one of) the detections was an adult male rusa deer? This structure allows for the possibility that adult male rusa deer may be present at a location, but will not always be detected there, even though hinds or juveniles are present. We expected the probability of detecting adult male rusa deer to increase in periods of greater adult male rusa deer activity. A range of models was fitted to the data to determine if parameters varied seasonally and whether rusa deer detection was different for those locations with and without adult male rusa deer. Season was included for overall occupancy and detection in all models. The 12 candidate multistate, multiseason occupancy models that we confronted with data are listed in S4 Table.

The camera trap data collected in summer 2011 were analysed using a two-species occupancy model [27, 28] to investigate whether rusa deer and red deer occupied similar locations and whether the covariates affecting occupancy and detection probabilities for each species were similar in summer. We used an approach comparable with that above to determine the relative importance of plant composition, of total, direct, and diffuse solar radiation, and of the three soil fertility covariates, although season was replaced by species as a factor. Species-level effects were retained in all models for both occupancy and detection. The 59 candidate two-species occupancy models that we confronted with data are listed in S5–S7 Tables.

All three types of models were fitted to the data using the software PRESENCE (available from http://www.mbr-pwrc.usgs.gov/software/presence.shtml), implemented via R [56] using specifically developed code. For each type of occupancy modelling used, we compared and ranked models using Akaike’s Information Criterion (AIC) [57]. Separate analyses were conducted to assess how solar, soil or plant composition covariates related to occupancy and detection; overall comparisons were made by comparing the AIC values from each of these analyses. Model averaging was used to account for model selection uncertainty when summarising the results of analyses [28, 57, 58]. Model averaging was applied at the scale of the occupancy and detection probabilities, which were estimated from each model rather than by determining model-averaged regression coefficients. This approach is preferable because it ensures the quantities being averaged have a consistent interpretation across models [28]. In plotting model-averaged occupancy and detection probabilities against a specific covariate it was necessary to specify values for the other covariates that were included in the set of models for which the results were being combined. In most cases, the observed median value was used for the other covariates, except for total solar radiation, which has to be the sum of the direct and diffuse solar radiation values used to create the plots.

## Results

### Scope of the data

**Fig 3:**
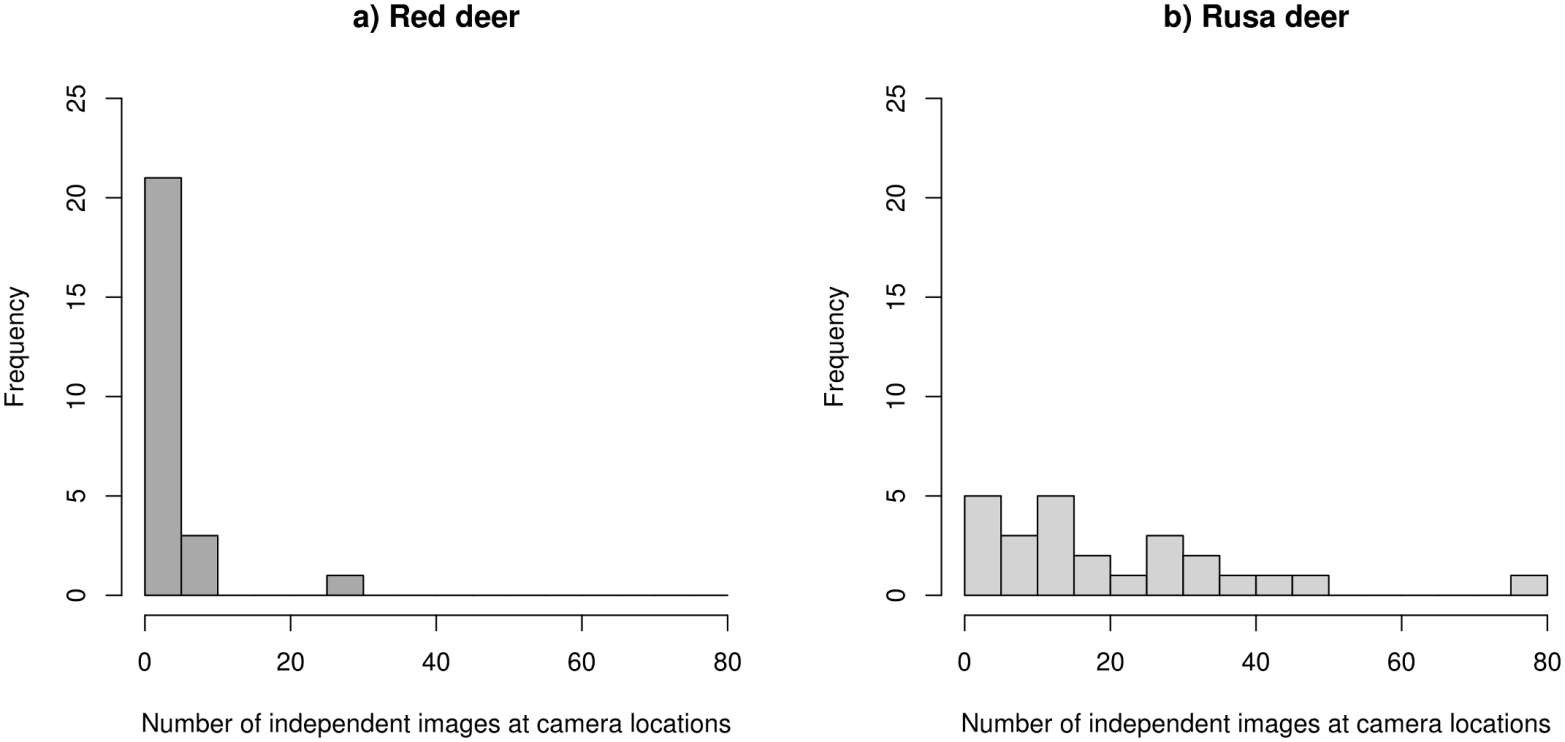
Frequency distributions of independent images of red deer (a) and rusa deer (b). Histograms are given for each deer species, with data from camera locations pooled across seasons and years (with bins 0–4, 5–9, etc.).

There were totals of 513 and 97 independent images of rusa deer and red deer, respectively (Table 1). Although there were ≥115 rusa deer images in each of the four seasons studied, red deer were not recorded on any images in winter 2010, and they were recorded on only 2 images in winter 2011. Red deer were recorded on 9 and 86 independent images in the summers of 2010 and 2011, respectively. The frequency of independent images per camera location (data pooled across seasons and years) were positively skewed for both deer species, although more so for red deer (Fig 3). Rusa deer images were recorded at 100% of the 25 camera locations at some point over the total period comprising the four seasons sampled, and red deer were recorded at 80% of the locations.

**Table 1:** Summary of the camera trap data for rusa deer and red deer by year and season.

	Winter 2010	Summer 2010	Winter 2011	Summer 2011
**Camera days**	2230	2313	2447	2753
**Rusa deer images** ^**a**^	115	129	116	153
**Rusa deer naïve occupancy**	0.72	0.76	0.84	0.92
**Red deer images** ^**a**^	0	9	2	86
**Red deer naïve occupancy**	0.00	0.16	0.08	0.76

Seasons were the winters of 2010 and 2011 and the austral summers of 2010/2011 (nominally “2010”) and 2011/2012 (nominally “2011”). The total number of camera days, number of images of each species, and naïve occupancy value (proportion of locations with at least one image during a season) are provided for each species for each season.

^a^Independent images (see Methods).

The winter total, direct and diffuse solar radiation (all in total mols m^-2^ day^-1^) varied between 0.3 and 7.4 (mean (±SE) of 1.3 ± 0.2), 0.0 and 5.0 (0.7 ± 0.2) and 0.2 and 2.4 (0.6 ± 0.1) across camera locations (data pooled across years), respectively. In this temperate oceanic climate, the summer total, direct and diffuse solar radiation overlapped with the winter values and varied between 1.1 and 14.4 (3.3 ± 0.4), 0.3 and 9.5 (1.9 ± 0.2) and 0.4 and 5.3 (1.4 ± 0.2) across camera locations, respectively. Mineral soil C:N ratio varied between 11 and 24 (14.9 ± 0.6), soil pH between 5.4 and 6.9 (6.2 ± 0.1) and available P between 3 and 41 μg g^-1^ (13.5 ± 1.9). Camera locations with low NMDS Axis 1 scores (Community 4; *n* = 9) were dominated by the trees *Hedycarya arborea* and *Melicytus ramiflorus*, the subcanopy shrub *Macropiper excelsum* and the fern *Polystichum neozelandicum*. The trees *Beilschmiedia tawa*, *Knightia excelsa* and *Melicytus ramiflorus* and the sedge *Uncinia uncinata* distinguished Community 5 (*n* = 6), which had higher NMDS Axis 1 scores than Community 4. Both these communities were unburnt forest with relatively low plant species richness (means of 11.2 ± 1.2 and 11.9 ± 0.8, respectively). Then, at even higher NMDS Axis 1 scores, *Knightia excelsa* dominated the canopy on previously burnt sites, sometimes also occurring with the tree *Weinmannia racemosa* and the understorey plant *Uncinia uncinata* (Community 2; *n* = 3). Finally, the highest scores were assigned to locations in Community 1 (*n* = 6) dominated by the tree *Kunzea ericoides*, found with *Uncinia uncinata* and *Uncinia rupestris*. Community 1 had relatively low canopy cover but high plant species richness (19.2 ± 1.0), with a diversity of shrubs (e.g. *Urtica ferox* and *Pseudopanax crassifolius*) and native (e.g. *Hydrocotyle moschata* and *Gnaphalium audax*) and exotic (e.g. *Hypochaeris radicata*, *Jacobaea vulgaris* and *Conyza canadensis*) herbs.

### Multiseason rusa deer occupancy models

**Fig 4:**
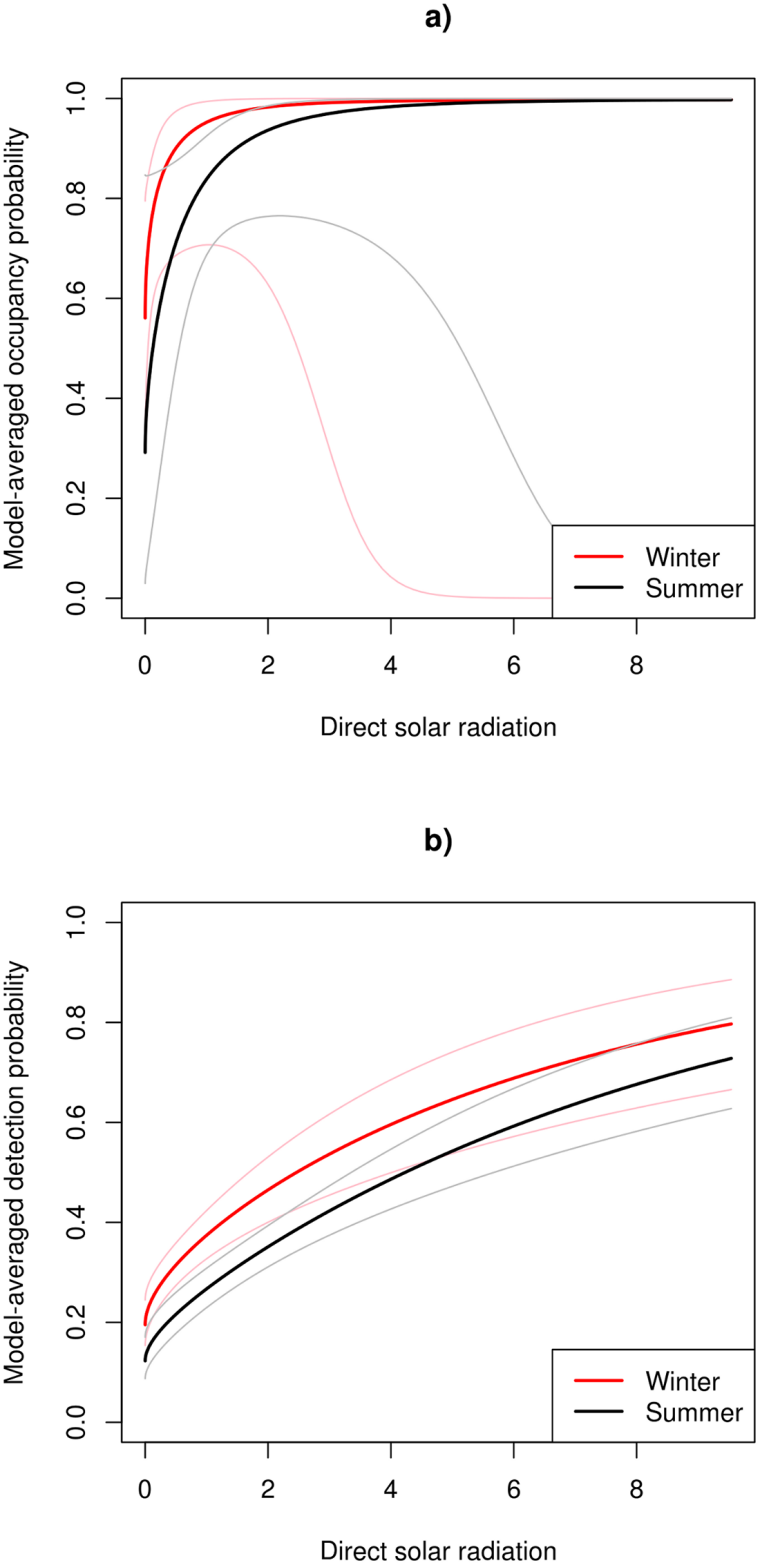
Seasonal model-averaged occupancy (a) and detection (b) probabilities for rusa deer versus direct solar radiation. Direct solar radiation (total mols m^-2^ day^-1^) refers to that transmitted through the canopy. Lighter lines indicate 95% confidence intervals.

Direct solar radiation appeared in the four highest-ranked solar radiation models and accounted for 0.99 of the model weights; hence, this was the best solar radiation predictor of occupancy and detection probabilities. Two of the four highest-ranked models involved an interaction term between season and direct solar radiation for occupancy or detection probabilities (Table 2), allowing the influence of warmth to be different in the two seasons. However, these models only had a combined model weight of 0.28 and 0.32 for occupancy and detection probabilities, respectively (Table 2). Model-averaged occupancy (Fig 4a) and detection (Fig 4b) probabilities both increased with direct solar radiation, and tended to be higher in winter than summer at the same level of solar radiation. The large increase in the confidence intervals for the model-averaged occupancy probability (Fig 4a) above a direct solar radiation value of 2 is due to a number of factors, including the fact that there were few observations with higher direct solar radiation values.

**Table 2:** Multiseason occupancy models for rusa deer camera trap data collected in winter and summer.

Occupancy	Detection	ΔAIC	*w*_*i*_	*K*	−2*LL*
**Direct, Diffuse and Total solar radiation (highest-ranked model AIC = 1473.48)**					
Season + Direct	Season + Direct + Number	0.00	0.48	7	1459.48
Season + Direct	Season × Direct + Number	1.48	0.23	8	1458.96
Season × Direct	Season + Direct + Number	1.89	0.19	8	1459.37
Season × Direct	Season × Direct + Number	3.33	0.09	9	1458.81
**Soil C:N ratio, P and pH (highest-ranked model AIC = 1558.00)**					
Season	Season + C:N ratio + Number	0.00	0.30	6	1546.00
Season	Season × C:N ratio + Number	1.99	0.11	7	1545.99
Season + C:N ratio	Season + C:N ratio + Number	2.00	0.11	7	1546.00
Season	Season + Number	3.14	0.06	5	1551.14
**Axis 1 (highest-ranked model AIC = 1527.70)**					
Season + Axis 1	Season + Axis 1 + Number	0.00	0.35	7	1513.70
Season + Axis 1	Season × Axis 1 + Number	0.74	0.24	8	1512.44
Season × Axis 1	Season + Axis 1 + Number	1.19	0.19	8	1512.89
Season × Axis 1	Season × Axis 1 + Number	1.44	0.17	9	1511.14

Year effects were not considered in models. Direct, diffuse and total solar radiation (each in total mols m^-2^ day^-1^), mineral soil percentage total carbon to percentage total nitrogen ratio (C:N ratio), Bray 2 available P (P, μg g^-1^) and pH, as well as non-metric multidimensional scaling axis 1 (Axis 1) scores, were each used, along with the number of camera operating days in a week (Number), as covariates in models for occupancy and detection. The relative difference in Akaike Information Criterion (ΔAIC), AIC model weight (*w*_*i*_), number of estimated parameters (*K*) and twice the negative log-likelihood value (−2*LL*) are provided. Only models with *w*_*i*_ > 0.05 are included.

Of the 59 multiseason occupancy models evaluated for rusa deer, the top-ranked solar radiation model was 84.52 AIC points better than the top-ranked soil-related model, and 54.22 AIC points better than the top-ranked plant composition model (Table 2). The AIC and model weights from a further multiseason occupancy model (details not tabulated) that included all possible combinations of the top-ranked solar radiation, soil fertility and plant composition covariates were 1473.48 and 0.48, respectively, for direct solar radiation alone, and 1476.91 and 0.10, respectively, for a combination of direct solar radiation, NMDS Axis 1 and mineral soil C:N ratio. This suggests that direct solar radiation largely subsumed the effects of other covariates.

### Multistate, multiseason adult male rusa deer occupancy models

**Fig 5:**
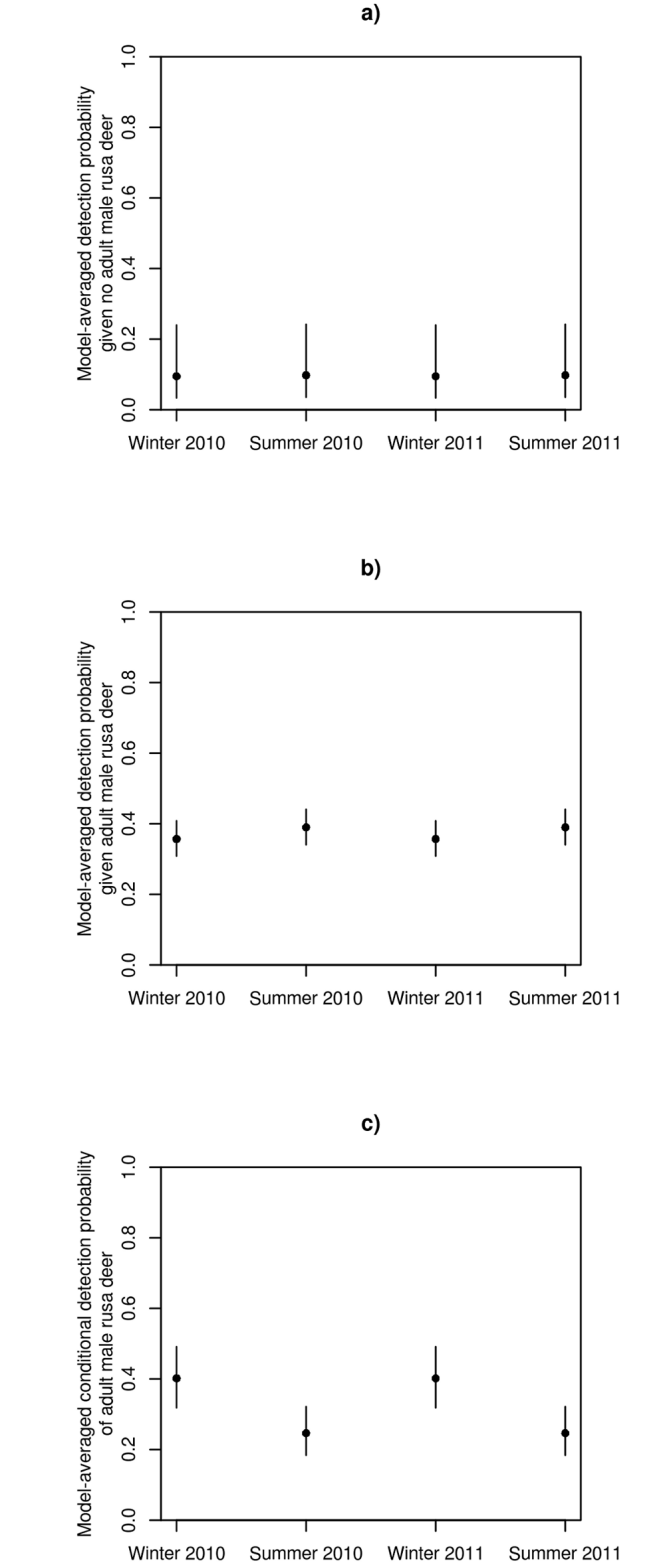
Model-averaged detection probabilities of rusa deer. The model-averaged detection probabilities of rusa deer at locations without adult male rusa deer (**a**), of rusa deer at locations with adult male rusa deer (**b**), and the conditional detection probability of adult male rusa deer given rusa deer have been detected at locations with adult male rusa deer (**c**) in each season and year sampled. Vertical lines indicate 95% confidence intervals.

Of the 12 multistate, multiseason occupancy models considered, there was greater evidence of seasonal variation in conditional detection probability than in conditional occupancy probability (Table 3). Our results also provide strong evidence that the detection probability of rusa deer differed depending on whether or not adult male rusa deer were detected (Table 3). Model-averaged estimates of the detection probability of rusa deer at locations without adult male rusa deer were lower than at locations with adult male rusa deer (Fig 5a and 5b). Also, given that rusa deer (male or female) were detected at a location, then the model-averaged detection probability of adult male rusa deer was higher in winter than in summer (Fig 5c).

**Table 3:** Multistate, multiseason models fitted to the rusa adult male camera trap data collected in winter and summer.

Occupancy	Conditional occupancy	Detection	Conditional detection	ΔAIC	*w*_*i*_	*K*	−2*LL*
Season	•	State + Season	Season	0.00	0.49	8	1970.34
Season	•	State × Season	Season	1.80	0.20	9	1970.14
Season	Season	State + Season	Season	1.83	0.20	9	1970.16
Season	Season	State × Season	Season	3.62	0.08	10	1969.96

Year effects were not considered in models. The conditional states for occupancy and detection probabilities in the model are: (1) no rusa deer; (2) rusa deer, but no rusa deer stags; and (3) rusa deer stags (and possibly other rusa deer). A “•” model indicates that the parameter is constant. The relative difference in Akaike Information Criterion (ΔAIC), AIC model weight (*w*_*i*_), number of estimated parameters (*K*) and twice the negative log-likelihood value (−2*LL*) are given. Only models with *w*_*i*_ > 0.05 are included. The AIC value for the highest-ranked model was 1986.34.

### Two-species rusa deer and red deer occupancy models

**Fig 6:**
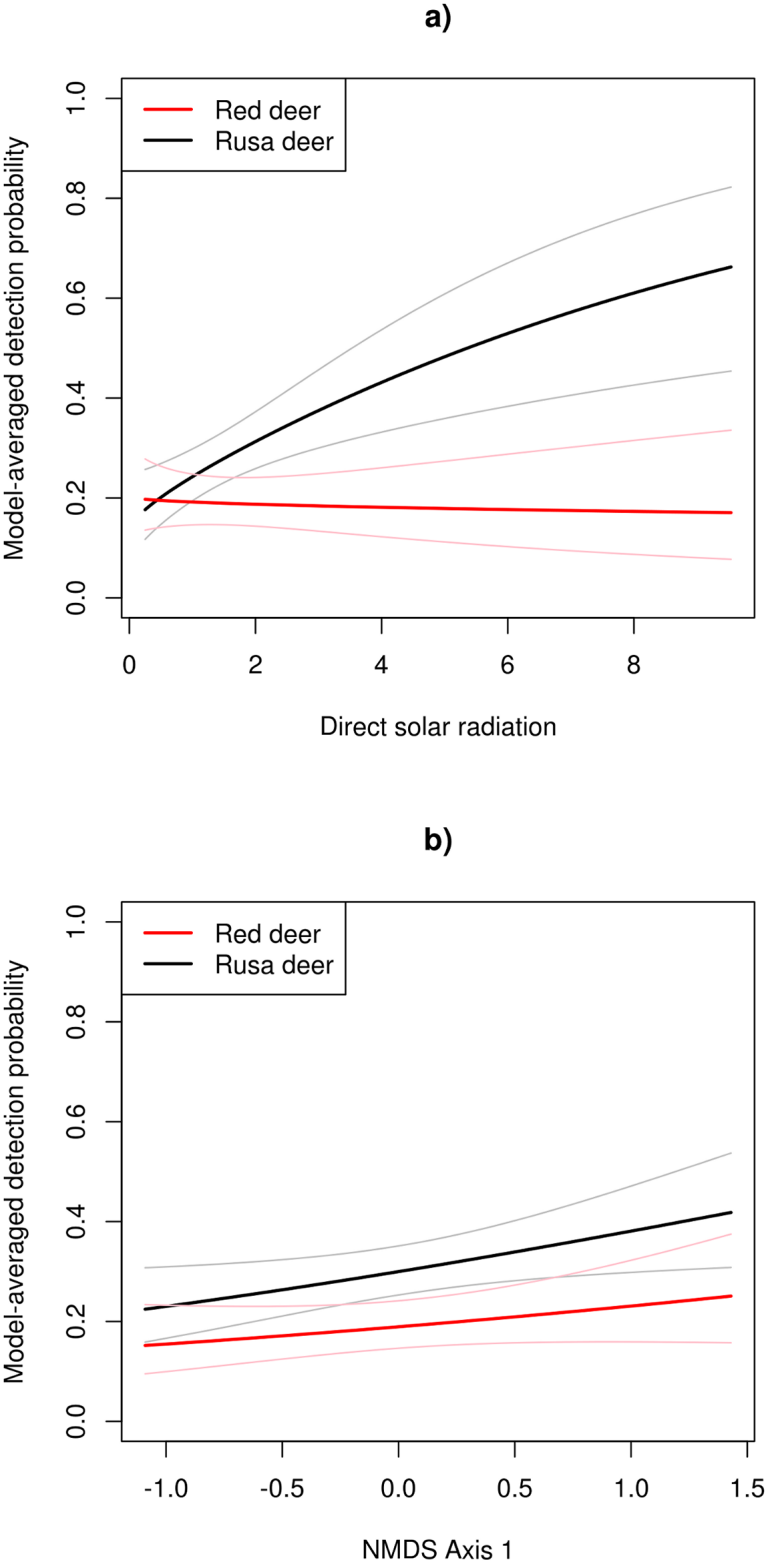
Model-averaged detection probability of rusa deer and red deer in summer 2011. Model-averaged detection probability of rusa deer and red deer in summer 2011 as a function of direct solar radiation (total mols m^-2^ day^-1^) transmitted through the canopy (**a**), and non-metric multidimensional scaling axis 1 (NMDS Axis 1) scores (**b**). Lighter lines indicate 95% confidence intervals.

When both deer species were relatively common, in summer 2011, the top-ranked warmth-related direct solar radiation model was 21.50 AIC points better than the top-ranked soil-related model and 18.37 points better than the top-ranked plant composition model (Table 4). These two-species model results are similar to those reported for rusa deer using the multiseason occupancy models (Table 2). Two of the four highest-ranked occupancy models did not include any of the solar radiation covariates (model weights total 0.63), indicating that occupancy was consistent with respect to our measure of warmth (Table 4). Occupancy probability was estimated to be higher for rusa deer than for red deer in the summer of 2011. The three highest-ranked models for detection probability and solar radiation all involved an interaction between species and direct solar radiation and had a combined model weight of 0.81 (Table 4). Detection probability of rusa deer increased with direct solar radiation from a similar level to that of red deer at low levels of direct solar radiation, but the detection probability of red deer was constant (Fig 6a). As a consequence, the detection probability of rusa deer at high levels of summer direct solar radiation was considerably higher than that of red deer.

**Table 4:** Two-species occupancy models for rusa deer and red deer camera trap data collected in summer 2011.

Occupancy	Detection	ΔAIC	*w*_*i*_	*K*	−2*LL*
**Direct, Diffuse and Total solar radiation (highest-ranked model AIC = AIC 772.77)**					
Species	Species × Direct + Number	0.00	0.47	7	758.77
Species + Direct	Species × Direct + Number	1.89	0.18	8	758.65
Species	Species × Total + Number	2.15	0.16	7	760.92
Species × Direct	Species × Direct + Number	3.58	0.08	9	758.35
**Soil C:N ratio, P and pH (highest-ranked model AIC = 794.27)**					
Species	Species × P + Number	0.00	0.18	7	780.27
Species + P	Species × P + Number	0.75	0.13	6	779.02
Species	Species + C:N ratio + Number	1.13	0.10	6	783.41
Species × P	Species × P + Number	1.68	0.08	9	777.96
Species	Species + P + Number	2.33	0.06	6	784.60
Species	Species + Number	2.33	0.06	5	786.60
**Axis 1 (highest-ranked model AIC = 791.14)**					
Species	Species + Axis 1 + Number	0.00	0.37	6	779.14
Species	Species × Axis 1 + Number	0.71	0.26	7	777.85
Species + Axis 1	Species + Axis 1 + Number	1.81	0.15	7	778.95
Species + Axis 1	Species × Axis 1 + Number	2.59	0.10	8	777.72
Species × Axis 1	Species + Axis 1 + Number	3.77	0.06	8	778.91

Species with direct, diffuse and total solar radiation (each in total mols m^-2^ day^-1^), mineral soil percentage total carbon to percentage total nitrogen ratio (C:N ratio), Bray 2 available P (P, μg g^-1^) and pH, as well as non-metric multidimensional scaling axis 1 (Axis 1) scores, were each used, along with the number of camera operating days in a week (Number), as covariates in models for occupancy and detection. The relative difference in Akaike Information Criterion (ΔAIC), AIC model weight (*w*_*i*_), number of estimated parameters (*K*) and twice the negative log-likelihood value (−2*LL*) are given. Only models with *w*_*i*_ > 0.05 are included.

The AIC and model weights from a further two-species occupancy model (details not tabulated) that included all possible combinations of the top-ranked solar radiation, soil fertility and plant composition covariates were 772.77 and 0.47, respectively, for direct solar radiation alone and 776.32 and 0.10, respectively, for a combination of direct solar radiation, NMDS Axis 1 and mineral soil available P. This again suggests that direct solar radiation largely subsumed the effects of the other covariates. This was unsurprising for plant composition because direct solar radiation was strongly correlated with NMDS Axis 1 scores (Pearson’s correlation = 0.61; *p* ≤ 0.001). Direct solar radiation was not, however, related to soil fertility covariates (Pearson’s correlation ≤ ±0.21; *p* = 0.24). There was a positive relationship between NMDS Axis 1 scores and detection probability, particularly for rusa deer (Fig 6b). Low detection probabilities were found in unburnt forest communities dominated by trees such as *Beilschmiedia tawa* and *Melicytus ramiflorus* (found at locations with low direct solar radiation), whereas high detection probabilities were found in burnt forest now dominated by *Knightia excelsa* and *Kunzea ericoides*, with high direct solar radiation.

## Discussion

Consistent with our hypothesis that the distribution of rusa deer in New Zealand is limited by low winter temperatures, rusa deer occupancy and detection probabilities were best predicted by, and increased with, total direct solar radiation. In the mountainous terrain of Te Urewera (Fig 1) the highest direct solar radiation occurs on those north-facing upper slopes and ridge crests that have a relatively open canopy, whereas the lowest solar radiation occurs on those south-facing lower slopes that have a relatively closed canopy. Habitat selection by rusa deer appears to be a response to an abiotic factor that achieves the radiative heat gain necessary, at least in part, for survival, particularly of juveniles [32]. Rusa deer have coarse, flattened guard hairs over much of the body, suitable for sun protection and dissipating tropical heat, whereas red deer have a thick undercoat with long rounded guard hairs designed to retain heat in temperate environments [31, 45, 59]. That rusa deer appear to seek radiant heat (i.e. selected among locations with relatively small-scale warmth difference) is the converse of moose (*Alces alces*) selecting for small-scale thermal shelters in boreal forest summers [8, 60]. In that situation, moose selected locations where the forest canopy structure offered shelter from thermal stress during warm periods [8]. Such small-scale habitat-fitness differences for large herbivores could have strong distributional and demographic implications for introduced and native species in the face of a changing climate [8, 21].

An unanticipated result was that red deer vacated the study area in both winters, even though red deer and rusa deer have been sympatric in the study area for over 50 years [35]. We expected that red deer would remain in the study area in winter, but be less influenced by solar radiation than rusa deer. Because tropical rusa deer do not face a lack of winter plant growth in their natural range, we might expect that they would need to exploit the best food sources in winter, whereas red deer have well-developed energy-saving strategies for harsh winter environments [61]. For example, red deer have low food intake and weight gain in winter compared with sambar deer (*Cervus unicolor*) [62], a tropical species closely related to rusa deer. Although we cannot exclude seasonal migration or exploitation competition as the reason why red deer were essentially absent from the study area in winter, our results suggest another plausible mechanism that could be responsible for the absence of red deer in winter. Habitat use and the level of activity by adult male rusa deer were greater only in winter at locations frequented by other rusa deer. While this on its own is unsurprising, given that winter includes the rusa deer rut [35], it is of significance because adult male rusa deer are viciously aggressive at that time [30, 36, 63]. Adult male rusa deer may persuade intercepted red deer to vacate the study area in winter. This would be exacerbated by the notable movement and range shifts exhibited by red deer [31]. We did not record any instances of adult male rusa deer being aggressive to red deer on our camera images in winter, but if behavioural interference was strong when it did occur, then it may only need to occur occasionally to be biologically important. It is of note that aggression by adult male rusa deer towards red deer in winter, and the movement of red deer out of habitat favoured by rusa deer, has been observed elsewhere in Te Urewera (e.g. T. Rua, personal observation). Adult male fallow deer (*Dama dama*) are aggressive towards a range of deer species in Europe [64, 65], and the resulting displacement of roe deer (*Capreolus capreolus*) by fallow deer in central Italy appears to be seasonal but unrelated to habitat [65], a result similar to our own.

Our results raise questions about the seasonal movements of red deer (in winter) and adult male rusa deer (in summer) within New Zealand forests. As the camera locations systematically sampled a small study area considered representative of the Ikawhenua Range, we do not know where red deer went during winter. In Norway, red deer undertake long seasonal migrations to exploit changing food resources [66], and similar movements may occur in New Zealand [17, 67]. The increased use and activity by adult male rusa deer during winter, relative to summer, suggests that this age–sex class resided elsewhere in summer. Adult male ungulates commonly segregate from females outside of the rutting season (i.e. for most of the year) (review in [68]) but, as for red deer, we do not know where adult male rusa deer went in summer. Understanding interactions between ungulate species is complicated by such seasonal movements, and is often progressively developed as results come to hand. There are two ways to progress the knowledge of seasonal movements. One is to conduct monitoring at fixed locations (e.g. camera traps) at a larger spatial scale (e.g. a 10 × 10 km study area) than we used. As our study may only be based upon a modest sample of individuals (although estimates of absolute deer densities are lacking for these forests) this approach would likely increase the number of individuals included. Monitoring at a larger spatial scale would be financially and logistically difficult to undertake in the rugged terrain of Te Urewera (Fig 1); moreover, it remains unclear what the appropriate scale is. Another way would be to track the movements (e.g. with GPS collars) of a random subsample of animals captured within the study area. Again, there would be financial and logistical challenges in capturing sufficient numbers of such animals for robust inference (review in [69]).

We hypothesised that if both red deer and rusa deer were present in summer, they would both select locations with an abundance of preferred browse plants. Those locations with high deer activity (high NMDS Axis 1 scores and, surprisingly, high direct solar radiation) were often dominated by canopy trees (e.g. *Kunzea ericoides* and *Knightia excelsa*) that are not preferred by browsing deer, whereas those locations with low deer activity (low NMDS Axis 1 scores and low direct solar radiation) were sometimes dominated by canopy trees, such as *Melicytus ramiflorus*, that are preferentially browsed by deer [70, 71]. Even though these are canopy trees, one might still expect deer to be active at these locations because they are known to feed on their fallen leaves [17], but this was not the case. The understorey (<2 m height) of locations with high deer activity, which tended to be in forest recovering from disturbance (e.g. Community 1), had instead a diversity of herb and grass species, many species of which are consumed by rusa deer, red deer or both [70, 72, 73]. Given that deer can select plants to consume with high foliar nutrient concentrations (e.g. of P and N) [20], it was surprising that plant composition (NMDS Axis 1) was unrelated to mineral soil available P (Pearson’s correlation = −0.21; *p* = 0.24) and significantly, but positively, related to mineral soil C:N ratio (Pearson’s correlation = 0.51; *p* ≤ 0.001). Our study was not designed to distinguish browse preference differences between these deer species, and hence we could not quantify the potential for exploitation competition. Elsewhere, sympatric introduced deer species have demonstrated different browse preferences at the same location due to, for example, sika deer having a different digestive morphology [14] and higher dietary versatility [74] than red deer.

## Conclusions

Our study shows the seasonal importance of both abiotic and biotic factors in determining large mammal site occupancy and detection probabilities at small spatial scales. In mountainous terrain, such as in our study area, topographic diversity leads to large variation in abiotic factors over small distances [38]. As expected, this is markedly so for solar radiation in the temperate zone, and this was the most important factor determining rusa deer site occupancy and detection probabilities in winter. This supports our hypothesis that the distribution of this tropical deer species is limited by low winter temperatures. An important biotic predictor was plant composition, which influenced the detection probability of both rusa deer and red deer in summer. Our results are also consistent with another biotic factor, behavioural interference, determining site occupancy by red deer in winter—albeit at a spatial scale larger than that of our study area. Thus, habitat use by coexisting tropical and temperate deer species in New Zealand’s temperate rainforests likely depends upon the interplay between thermoregulatory and behavioural traits of the deer species and the abiotic and biotic characteristics of the ecosystem.

## Supporting Information

S1 Dataset Data input file for the multiseason rusa deer occupancy models analysed using PRESENCE. (PAO)

S2 Dataset Data input file for the multistate, multiseason adult male rusa deer occupancy models analysed using PRESENCE. (PAO)

S3 Dataset Data input file for the two-species rusa deer and red deer occupancy models analysed using PRESENCE. (PAO)

S1 Table Model selection summary for the 25 warmth-related solar radiation models fitted to the rusa deer camera trap data collected in winter and summer. (DOCX)

S2 Table Model selection summary for the 25 soil-related models fitted to the rusa deer camera trap data collected in winter and summer. (DOCX)

S3 Table Model selection summary for the nine plant composition–related models fitted to the rusa deer camera trap data collected in winter and summer. (DOCX)

S4 Table Model selection summary for the 12 models fitted to the adult male rusa deer camera trap data collected in winter and summer. (DOCX)

S5 Table Model selection summary for the 25 temperature-related models fitted to the rusa deer and red deer camera trap data collected in summer 2011. (DOCX)

S6 Table Model selection summary for the 25 soil-related models fitted to the rusa deer and red deer camera trap data collected in summer 2011. (DOCX)

S7 Table Model selection summary for the nine plant composition–related models fitted to the rusa deer and red deer camera trap data collected in summer 2011. (DOCX)

S1 Text Metadata for the occupancy model data input files analysed using the software PRESENCE. (DOCX)
