# Supplementary material for: Solar Radiation Determines Site Occupancy of Coexisting Tropical and Temperate Deer Species Introduced to New Zealand Forests
Source: PLoS One. 2015 Jun 10;10(6):e0128924. doi: 10.1371/journal.pone.0128924 (PMC4465677; doi:10.1371/journal.pone.0128924)
Supplement: S1 Table — (DOCX) [file pone.0128924.s004.docx]

**S1 Table. Model selection summary for the 25 warmth-related solar radiation models ﬁtted to the rusa deer camera trap data collected in winter and summer.**

| **Occupancy** | **Detection** | **ΔAIC** | ***w_i_*** | ***K*** | **−2*LL*** |
| --- | --- | --- | --- | --- | --- |
| Season + Direct | Season + Direct + Number | 0.00 | 0.48 | 7 | 1,459.48 |
| Season + Direct | Season × Direct + Number | 1.48 | 0.23 | 8 | 1,458.96 |
| Season × Direct | Season + Direct + Number | 1.89 | 0.19 | 8 | 1,459.37 |
| Season × Direct | Season × Direct + Number | 3.33 | 0.09 | 9 | 1,458.81 |
| Season | Season + Direct + Number | 8.02 | 0.01 | 6 | 1,469.49 |
| Season | Season × Direct + Number | 9.73 | 0.00 | 7 | 1,469.21 |
| Season + Total | Season + Total + Number | 17.02 | 0.00 | 7 | 1,476.50 |
| Season × Total | Season + Total + Number | 17.44 | 0.00 | 8 | 1,474.92 |
| Season + Total | Season × Total + Number | 18.33 | 0.00 | 8 | 1,475.81 |
| Season × Total | Season × Total + Number | 18.69 | 0.00 | 9 | 1,474.17 |
| Season | Season + Total + Number | 24.30 | 0.00 | 6 | 1,485.78 |
| Season | Season × Total + Number | 25.70 | 0.00 | 7 | 1,485.18 |
| Season + Diffuse | Season × Diffuse + Number | 48.57 | 0.00 | 8 | 1,506.05 |
| Season × Diffuse | Season × Diffuse + Number | 50.30 | 0.00 | 9 | 1,505.78 |
| Season | Season × Diffuse + Number | 50.47 | 0.00 | 7 | 1,509.95 |
| Season + Diffuse | Season + Diffuse + Number | 50.48 | 0.00 | 7 | 1,509.96 |
| Season × Diffuse | Season + Diffuse + Number | 52.22 | 0.00 | 8 | 1,509.70 |
| Season | Season + Diffuse + Number | 52.36 | 0.00 | 6 | 1,513.84 |
| Season + Direct | Season + Number | 76.75 | 0.00 | 6 | 1,538.23 |
| Season × Direct | Season + Number | 78.30 | 0.00 | 7 | 1,537.78 |
| Season + Total | Season + Number | 79.44 | 0.00 | 6 | 1,540.92 |
| Season × Total | Season + Number | 79.53 | 0.00 | 7 | 1,539.01 |
| Season + Diffuse | Season + Number | 85.62 | 0.00 | 6 | 1,547.10 |
| Season × Diffuse | Season + Number | 87.36 | 0.00 | 7 | 1,546.84 |
| Season | Season + Number | 87.66 | 0.00 | 5 | 1,551.14 |

Year effects were not considered in models. Direct, diffuse and total solar radiation were used, along with the number of camera operating days in a week (Number), as covariates in models for occupancy and detection. Also given are the relative diﬀerence in Akaike’s Information Criterion (ΔAIC), AIC model weight (*w_i_*), number of parameters in the model (*K*) and twice the negative log-likelihood value (*−2LL*). The AIC value for the top-ranked model was 1473.48.
