# Supplementary material for: Solar Radiation Determines Site Occupancy of Coexisting Tropical and Temperate Deer Species Introduced to New Zealand Forests
Source: PLoS One. 2015 Jun 10;10(6):e0128924. doi: 10.1371/journal.pone.0128924 (PMC4465677; doi:10.1371/journal.pone.0128924)
Supplement: S2 Table — (DOCX) [file pone.0128924.s005.docx]

**S2 Table. Model selection summary for the 25 soil-related models ﬁtted to the rusa deer camera trap data collected in winter and summer.**

| **Occupancy** | **Detection** | **ΔAIC** | ***w_i_*** | ***K*** | **−2*LL*** |
| --- | --- | --- | --- | --- | --- |
| Season | Season + C:N ration + Number | 0.00 | 0.30 | 6 | 1,546.00 |
| Season | Season × C:N ratio + Number | 1.99 | 0.11 | 7 | 1,545.99 |
| Season + C:N ratio | Season + C:N ratio + Number | 2.00 | 0.11 | 7 | 1,546.00 |
| Season | Season + Number | 3.14 | 0.06 | 5 | 1,551.14 |
| Season × C:N ratio | Season + C:N ratio + Number | 3.40 | 0.05 | 8 | 1,545.40 |
| Season + pH | Season + Number | 3.80 | 0.04 | 6 | 1,549.80 |
| Season + C:N ratio | Season × C:N ratio + Number | 3.99 | 0.04 | 8 | 1,545.99 |
| Season | Season + Bray + Number | 4.16 | 0.04 | 6 | 1,550.16 |
| Season + P | Season + Number | 4.49 | 0.03 | 6 | 1,550.49 |
| Season × pH | Season + Number | 4.75 | – | 7 | 1,548.75 |
| Season | Season + pH + Number | 4.87 | 0.03 | 6 | 1,550.87 |
| Season | Season × Bray + Number | 4.93 | 0.03 | 7 | 1,548.92 |
| Season + C:N ratio | Season + Number | 5.13 | 0.02 | 6 | 1,551.13 |
| Season × P | Season + Number | 5.25 | 0.02 | 7 | 1,549.25 |
| Season × C:N ratio | Season × C:N ratio + Number | 5.39 | 0.02 | 9 | 1,545.39 |
| Season + P | Season + Bray + Number | 5.46 | 0.02 | 7 | 1,549.46 |
| Season + pH | Season + pH + Number | 5.56 | 0.02 | 7 | 1,549.56 |
| Season × P | Season + Bray + Number | 6.23 | 0.01 | 8 | 1,548.23 |
| Season + P | Season × Bray + Number | 6.25 | 0.01 | 8 | 1,548.25 |
| Season × pH | Season + pH + Number | 6.52 | – | 8 | 1,548.52 |
| Season × C:N ratio | Season + Number | 6.54 | 0.01 | 7 | 1,550.53 |
| Season | Season × pH + Number | 6.78 | 0.01 | 7 | 1,550.78 |
| Season × P | Season × Bray + Number | 6.94 | 0.01 | 9 | 1,546.94 |
| Season + pH | Season × pH + Number | 7.47 | 0.01 | 8 | 1,549.47 |
| Season × pH | Season × pH + Number | 8.41 | – | 9 | 1,548.41 |

Year effects were not considered in models. Mineral soil percentage total carbon to total nitrogen ratio (C:N ratio), Bray 2 available phosphorus (P) and pH were used, along with the number of camera operating days in a week (Number), as covariates in models for occupancy and detection. Also given are the relative diﬀerence in Akaike’s Information Criterion (ΔAIC), AIC model weight (*w_i_*), number of parameters in the model (*K*) and twice the negative log-likelihood value (*−2LL*). Models with no model weight value were excluded from the model averaging. The AIC value for the top-ranked model was 1558.00.
