# Supplementary material for: Solar Radiation Determines Site Occupancy of Coexisting Tropical and Temperate Deer Species Introduced to New Zealand Forests
Source: PLoS One. 2015 Jun 10;10(6):e0128924. doi: 10.1371/journal.pone.0128924 (PMC4465677; doi:10.1371/journal.pone.0128924)
Supplement: S3 Table — (DOCX) [file pone.0128924.s006.docx]

**S3 Table. Model selection summary for the nine plant composition–related models ﬁtted to the rusa deer camera trap data collected in winter and summer.**

| **Occupancy** | **Detection** | **ΔAIC** | ***w_i_*** | ***K*** | **−2*LL*** |
| --- | --- | --- | --- | --- | --- |
| Season + Axis 1 | Season + Axis 1 + Number | 0.00 | 0.35 | 7 | 1,513.70 |
| Season + Axis 1 | Season × Axis 1 + Number | 0.74 | 0.24 | 8 | 1,512.44 |
| Season × Axis 1 | Season + Axis 1 + Number | 1.19 | 0.19 | 8 | 1,512.89 |
| Season × Axis 1 | Season × Axis 1 + Number | 1.44 | 0.17 | 9 | 1,511.14 |
| Season | Season + Axis 1 + Number | 4.50 | 0.04 | 6 | 1,520.20 |
| Season | Season × Axis 1 + Number | 5.58 | 0.02 | 7 | 1,519.29 |
| Season × Axis 1 | Season + Number | 26.49 | 0.00 | 7 | 1,540.19 |
| Season + Axis 1 | Season + Number | 26.55 | 0.00 | 6 | 1,542.25 |
| Season | Season + Number | 33.44 | 0.00 | 5 | 1,551.14 |

Year effects were not considered in models. Non-metric multidimensional scaling axis 1 (Axis 1) scores were used, along with the number of camera operating days in a week (Number), as covariates in models for occupancy and detection. Also given are the relative diﬀerence in Akaike’s Information Criterion (ΔAIC), AIC model weight (*w_i_*), number of parameters in the model (*K*) and twice the negative log-likelihood value (*−2LL*). Models with no model weight value were excluded from the model averaging. The AIC value for the top-ranked model was 1527.70.
