# Supplementary material for: Solar Radiation Determines Site Occupancy of Coexisting Tropical and Temperate Deer Species Introduced to New Zealand Forests
Source: PLoS One. 2015 Jun 10;10(6):e0128924. doi: 10.1371/journal.pone.0128924 (PMC4465677; doi:10.1371/journal.pone.0128924)
Supplement: S4 Table — (DOCX) [file pone.0128924.s007.docx]

**S4 Table. Model selection summary for the 12 models ﬁtted to the adult male rusa deer camera trap data collected in winter and summer.**

| **Occupancy** | **Conditional occupancy** | **Detection** | **Conditional detection** | **ΔAIC** | ***w_i_*** | ***K*** | **−2*LL*** |
| --- | --- | --- | --- | --- | --- | --- | --- |
| Season | • | State + Season | Season | 0.00 | 0.49 | 8 | 1,970.34 |
| Season | • | State × Season | Season | 1.80 | 0.20 | 9 | 1,970.14 |
| Season | Season | State + Season | Season | 1.83 | 0.20 | 9 | 1,970.16 |
| Season | Season | State × Season | Season | 3.62 | 0.08 | 10 | 1,969.96 |
| Season | • | State + Season | • | 7.45 | 0.01 | 7 | 1,979.78 |
| Season | • | State × Season | • | 8.56 | 0.01 | 8 | 1,978.89 |
| Season | Season | State + Season | • | 8.61 | 0.01 | 8 | 1,978.94 |
| Season | Season | State × Season | • | 10.16 | 0.00 | 9 | 1,978.50 |
| Season | Season | Season | • | 20.37 | 0.00 | 7 | 1,992.71 |
| Season | Season | Season | Season | 21.19 | 0.00 | 8 | 1,991.53 |
| Season | • | Season | Season | 23.48 | 0.00 | 7 | 1,995.81 |
| Season | • | Season | • | 25.53 | 0.00 | 6 | 1,999.87 |

Year effects were not considered in models. A “•” model indicates that the parameter is constant. The conditional states for occupancy and detection probabilities used in the model are: (1) no rusa deer; (2) rusa deer, but no rusa deer stags; and (3) rusa deer stags (and possibly other rusa deer). Also given are the relative diﬀerence in Akaike’s Information Criterion (ΔAIC), AIC model weight (*w_i_*), number of parameters in the model (*K*) and twice the negative log-likelihood value (*−2LL*). The AIC value for the top-ranked model was 1,986.34.
