# Supplementary material for: Solar Radiation Determines Site Occupancy of Coexisting Tropical and Temperate Deer Species Introduced to New Zealand Forests
Source: PLoS One. 2015 Jun 10;10(6):e0128924. doi: 10.1371/journal.pone.0128924 (PMC4465677; doi:10.1371/journal.pone.0128924)
Supplement: S5 Table — (DOCX) [file pone.0128924.s008.docx]

**S5 Table. Model selection summary for the 25 temperature-related models ﬁtted to the rusa deer and red deer camera trap data collected in summer 2011.**

| **Occupancy** | **Detection** | **ΔAIC** | ***w_i_*** | ***K*** | **−2*LL*** |
| --- | --- | --- | --- | --- | --- |
| Species | Species × Direct + Number | 0.00 | 0.47 | 7 | 758.77 |
| Species + Direct | Species × Direct + Number | 1.89 | 0.18 | 8 | 758.65 |
| Species | Species × Total + Number | 2.15 | 0.16 | 7 | 760.92 |
| Species × Direct | Species × Direct + Number | 3.58 | 0.08 | 9 | 758.35 |
| Species + Total | Species × Total + Number | 3.99 | 0.06 | 8 | 760.76 |
| Species × Total | Species × Total + Number | 5.71 | 0.03 | 9 | 760.48 |
| Species | Species × Diffuse + Number | 8.91 | 0.01 | 7 | 767.68 |
| Species + Diffuse | Species × Diffuse + Number | 10.47 | 0.00 | 8 | 767.24 |
| Species | Species + Direct + Number | 10.82 | 0.00 | 6 | 771.59 |
| Species | Species + Total + Number | 11.23 | 0.00 | 6 | 771.99 |
| Species × Diffuse | Species × Diffuse + Number | 12.40 | 0.00 | 9 | 767.17 |
| Species + Direct | Species + Direct + Number | 12.79 | 0.00 | 7 | 771.56 |
| Species + Total | Species + Total + Number | 13.11 | 0.00 | 7 | 771.88 |
| Species | Species + Diffuse + Number | 13.41 | 0.00 | 6 | 774.18 |
| Species × Direct | Species + Direct + Number | 14.30 | 0.00 | 8 | 771.06 |
| Species × Total | Species + Total + Number | 14.75 | 0.00 | 8 | 771.51 |
| Species + Diffuse | Species + Diffuse + Number | 15.01 | 0.00 | 7 | 773.77 |
| Species × Diffuse | Species + Diffuse + Number | 16.92 | 0.00 | 8 | 773.69 |
| Species | Species + Number | 23.83 | 0.00 | 5 | 786.60 |
| Species + Diffuse | Species + Number | 25.36 | 0.00 | 6 | 786.12 |
| Species + Total | Species + Number | 25.64 | 0.00 | 6 | 786.40 |
| Species + Direct | Species + Number | 25.70 | 0.00 | 6 | 786.47 |
| Species × Diffuse | Species + Number | 27.28 | 0.00 | 7 | 786.05 |
| Species × Total | Species + Number | 27.31 | 0.00 | 7 | 786.07 |
| Species × Direct | Species + Number | 27.32 | 0.00 | 7 | 786.09 |

Direct, diffuse and total solar radiation, along with the number of camera operating days in a week (Number) and species, were used as covariates in models for occupancy and detection. Also given are the relative diﬀerence in Akaike’s Information Criterion (ΔAIC), AIC model weight (*w_i_*), number of parameters in the model (*K*) and twice the negative log-likelihood value (*−2LL*). The AIC value for the top-ranked model was 772.77.
