# Supplementary material for: Solar Radiation Determines Site Occupancy of Coexisting Tropical and Temperate Deer Species Introduced to New Zealand Forests
Source: PLoS One. 2015 Jun 10;10(6):e0128924. doi: 10.1371/journal.pone.0128924 (PMC4465677; doi:10.1371/journal.pone.0128924)
Supplement: S6 Table — (DOCX) [file pone.0128924.s009.docx]

**S6 Table. Model selection summary for the 25 soil-related models ﬁtted to the rusa deer and red deer camera trap data collected in summer 2011.**

| **Occupancy** | **Detection** | **ΔAIC** | ***w_i_*** | ***K*** | **−2*LL*** |
| --- | --- | --- | --- | --- | --- |
| Species | Species × P + Number | 0.00 | 0.18 | 7 | 780.27 |
| Species + P | Species × P + Number | 0.75 | 0.13 | 8 | 779.02 |
| Species | Species + C:N ratio + Number | 1.13 | 0.10 | 6 | 783.41 |
| Species × P | Species × P + Number | 1.68 | 0.08 | 9 | 777.96 |
| Species | Species + P + Number | 2.33 | 0.06 | 6 | 784.60 |
| Species | Species + Number | 2.33 | 0.06 | 5 | 786.60 |
| Species | Species × C:N ratio + Number | 2.48 | 0.05 | 7 | 782.75 |
| Species + C:N ratio | Species + C:N ratio + Number | 2.80 | 0.05 | 7 | 783.07 |
| Species + P | Species + P + Number | 2.81 | 0.05 | 7 | 783.09 |
| Species + P | Species + Number | 3.00 | 0.04 | 6 | 785.27 |
| Species × pH | Species + Number | 3.25 | 0.04 | 7 | 783.52 |
| Species + pH | Species + Number | 3.48 | – | 6 | 785.75 |
| Species + C:N ratio | Species × C:N ratio + Number | 3.66 | 0.03 | 8 | 781.94 |
| Species | Species × pH + Number | 3.85 | – | 7 | 784.13 |
| Species × P | Species + P + Number | 3.90 | 0.03 | 8 | 782.18 |
| Species + pH | Species × pH + Number | 3.96 | – | 8 | 782.24 |
| Species | Species + pH + Number | 3.97 | 0.03 | 6 | 786.24 |
| Species × P | Species + Number | 4.01 | 0.02 | 7 | 784.28 |
| Species + C:N ratio | Species + Number | 4.27 | 0.02 | 6 | 786.55 |
| Species × C:N ratio | Species + C:N ratio + Number | 4.69 | 0.02 | 8 | 782.96 |
| Species × pH | Species × pH + Number | 4.75 | – | 9 | 781.02 |
| Species × pH | Species + pH + Number | 4.87 | 0.02 | 8 | 783.15 |
| Species + pH | Species + pH + Number | 4.91 | – | 7 | 785.18 |
| Species × C:N ratio | Species × C:N ratio + Number | 4.99 | – | 9 | 781.26 |
| Species × C:N ratio | Species + Number | 6.27 | 0.01 | 7 | 786.55 |

Mineral soil percentage total carbon to total nitrogen ratio (C:N ratio), Bray 2 available phosphorus (P) and pH were used, along with the number of camera operating days in a week (Number) and species, as covariates in models for occupancy and detection. Also given are the relative diﬀerence in Akaike’s Information Criterion (ΔAIC), AIC model weight (*w_i_*), number of parameters in the model (*K*) and twice the negative log-likelihood value (*−2LL*). The AIC value for the top-ranked model was 794.27.
