# Supplementary material for: Solar Radiation Determines Site Occupancy of Coexisting Tropical and Temperate Deer Species Introduced to New Zealand Forests
Source: PLoS One. 2015 Jun 10;10(6):e0128924. doi: 10.1371/journal.pone.0128924 (PMC4465677; doi:10.1371/journal.pone.0128924)
Supplement: S7 Table — (DOCX) [file pone.0128924.s010.docx]

**S7 Table.** **Model selection summary for the nine plant composition–related models ﬁtted to the rusa deer and red deer camera trap data collected in summer 2011.**

| **Occupancy** | **Detection** | **ΔAIC** | ***w_i_*** | ***K*** | **−2*LL*** |
| --- | --- | --- | --- | --- | --- |
| Species | Species + Axis 1 + Number | 0.00 | 0.37 | 6 | 779.14 |
| Species | Species × Axis 1 + Number | 0.71 | 0.26 | 7 | 777.85 |
| Species + Axis 1 | Species + Axis 1 + Number | 1.81 | 0.15 | 7 | 778.95 |
| Species + Axis 1 | Species × Axis 1 + Number | 2.59 | 0.10 | 8 | 777.72 |
| Species × Axis 1 | Species + Axis 1 + Number | 3.77 | 0.06 | 8 | 778.91 |
| Species × Axis 1 | Species × Axis 1 + Number | 4.57 | 0.04 | 9 | 777.71 |
| Species | Species + Number | 5.46 | 0.02 | 5 | 786.60 |
| Species + Axis 1 | Species + Number | 7.39 | 0.01 | 6 | 786.53 |
| Species × Axis 1 | Species + Number | 9.38 | 0.00 | 7 | 786.52 |

Non-metric multidimensional scaling axis 1 (Axis 1) scores were used, along with the number of camera operating days in a week (Number) and species, as covariates in models for occupancy and detection. Also given are the relative diﬀerence in Akaike’s Information Criterion (ΔAIC), AIC model weight (*w_i_*), number of parameters in the model (*K*) and twice the negative log-likelihood value (*−2LL*). Models with no model weight value were excluded from the model averaging. The AIC value for the top-ranked model was 791.14.
