# Supplementary material for: Solar Radiation Determines Site Occupancy of Coexisting Tropical and Temperate Deer Species Introduced to New Zealand Forests
Source: PLoS One. 2015 Jun 10;10(6):e0128924. doi: 10.1371/journal.pone.0128924 (PMC4465677; doi:10.1371/journal.pone.0128924)
Supplement: S1 Text — (DOCX) [file pone.0128924.s011.docx]

**S8 Text. Metadata for the occupancy model data input files analysed using the software PRESENCE.**

1. Metadata for the multiseason rusa deer occupancy models

The data input file for the multiseason rusa deer occupancy models analysed using PRESENCE is given in S9_Dataset.pao. The Metadata for this data input file is described below.

Number of locations = 25.

59 weeks surveyed in total over 4 seasons. Number of weeks surveyed per season was 14, 14, 15 and16 for the winter of 2010, summer of 2010, winter of 2011, and summer of 2011 respectively.

A week surveyed was the detection/nondetection of rusa deer within a 7-day period. Note cameras may not be operational at all sites for all 7 days.

Location covariates

sqrt_DirW10, sqrt_DirS10, sqrt_DirW11, sqrt_DirS11 = square root of direct solar radiation for the winter of 2010, summer of 2010, winter of 2011, and summer of 2011 respectively.

sqrt_DifW10, sqrt_DifS10, sqrt_DifW11, sqrt_DifS11 = square root of diffuse solar radiation for the winter of 2010, summer of 2010, winter of 2011, and summer of 2011 respectively.

sqrt_TotW10, sqrt_TotS10, sqrt_TotW11, sqrt_TotS11 = square root of total solar radiation for the winter of 2010, summer of 2010, winter of 2011, and summer of 2011 respectively.

ln_pH = log(soil pH).

ln_CN_rat = log(soil carbon-nitrogen ratio).

ln_P_bray = log(available soil phosphorous from Bray test).

NMDS = non-metric multi-dimensional scaling axis 1 score from plant species compositional data.

Survey covariates

Num_Surv = number of days camera was operational in a weekly survey period (7 days).

1. Multistate, multiseason adult male rusa deer occupancy models

The data input file for the multistate, multiseason adult male rusa deer occupancy models analysed using PRESENCE is given in S10_Dataset.pao. The Metadata for this data input file is described below.

Number of locations = 25.

59 weeks surveyed in total over 4 seasons. Number of weeks surveyed per season was 14, 14, 15 and 16 for the winter of 2010, summer of 2010, winter of 2011, and summer of 2011 respectively.

A week surveyed was the detection/nondetection of rusa deer within a 7-day period. Note cameras may not be operational at all sites for all 7 days.

Weekly survey results are coded as:

0 = no detection.

1 = detection of adult female rusa deer or juvenile rusa deer (i.e. no adult male rusa deer)

2 = detection of at least 1 adult male rusa deer (and possibly other rusa deer as well)

Location covariates

None.

Survey covariates

Num_Surv = number of days camera was operational in a weekly survey period (7 days).

1. Two-species rusa deer and red deer occupancy models

The data input file for the two-species rusa deer and red deer occupancy models analysed using PRESENCE is given in S11_Dataset.pao. The Metadata for this data input file is described below.

Number of locations = 25.

Rows 1-25 of data, and covariate values, relate to rusa deer data, and rows 25-50 of data, and covariate values, relate to red deer data.

16 weeks were surveyed in 1 season (summer 2011).

A week surveyed was the detection/nondetection of deer within a 7-day period. Note cameras may not be operational at all sites for all 7 days.

Location covariates

sqrt_DirS11 = square root of direct solar radiation for the summer of 2011.

sqrt_DifS11 = square root of diffuse solar radiation for the summer of 2011.

sqrt_TotS11 = square root of total solar radiation for the summer of 2011.

ln_pH = log(soil pH)

ln_CN_rat = log(soil carbon-nitrogen ratio)

ln_P_bray = log(available soil phosphorous from Bray test)

NMDS = non-metric mult-dimensional scaling axis 1 score from plant species compositional data.

Survey covariates

Num_Surv = number of days camera was operational in a weekly survey period (7 days).
